# Studies on Polybenzimidazole and Methanesulfonate Protic-Ionic-Liquids-Based Composite Polymer Electrolyte Membranes

**DOI:** 10.3390/polym15132821

**Published:** 2023-06-26

**Authors:** Arfat Anis, Manawwer Alam, Abdullah Alhamidi, Ravindra Kumar Gupta, Mohammad Tariq, Saeed M. Al-Zahrani

**Affiliations:** 1 SABIC Polymer Research Center (SPRC), Chemical Engineering Department, King Saud University, Riyadh 11421, Saudi Arabia; akfhk90@hotmail.com (A.A.); szahrani@ksu.edu.sa (S.M.A.-Z.); 2 Department of Chemistry, College of Science, King Saud University, Riyadh 11451, Saudi Arabia; maalam@ksu.edu.sa; 3 King Abdullah Institute for Nanotechnology, King Saud University, Riyadh 11451, Saudi Arabia; rgupta@ksu.edu.sa; 4 LAQV, REQUIMTE, Department of Chemistry, NOVA School of Science and Technology, NOVA University Lisbon, 2829-516 Caparica, Portugal; tariq@fct.unl.pt

**Keywords:** protic ionic liquids polybenzimidazole, polymer electrolyte membrane, thermal analysis, ionic conductivity, FTIR spectroscopy, NMR analysis

## Abstract

In the present work, different methanesulfonate-based protic ionic liquids (PILs) were synthesized and their structural characterization was performed using FTIR, ^1^H, and ^13^C NMR spectroscopy. Their thermal behavior and stability were studied using DSC and TGA, respectively, and EIS was used to study the ionic conductivity of these PILs. The PIL, which was diethanolammonium-methanesulfonate-based due to its compatibility with polybenzimidazole (PBI) to form composite membranes, was used to prepare proton-conducting polymer electrolyte membranes (PEMs) for prospective high-temperature fuel cell application. The prepared PEMs were further characterized using FTIR, DSC, TGA, SEM, and EIS. The FTIR results indicated good interaction among the PEM components and the DSC results suggested good miscibility and a plasticizing effect of the incorporated PIL in the PBI polymer matrix. All the PEMs showed good thermal stability and good proton conductivity for prospective high-temperature fuel cell application.

## 1. Introduction

Polymer electrolyte membrane fuel cells (PEMFCs) are a promising and viable alternative energy source for stationary, vehicular, and portable applications due to their efficient and direct conversion of the chemical energy of hydrogen fuel into electrical energy, with pure water as the only by-product of the process. These fuel cells use proton exchange membranes as electrolytes to efficiently transport the protons from the anode to the cathode without allowing any fuel crossover through the membranes. Low-temperature PEMFCs using perfluorosulfonic acid ionomers (Nafion) as electrolytes have been widely used for a long time. These membranes require hydration for proton conduction and, hence, cannot be used at temperatures above 80 °C. In contrast, high-temperature PEM fuel cells, which normally are operated at higher temperatures between 120 and 200 °C, are more attractive since they show improved electrode kinetics, improved resistance to catalyst poisoning by carbon monoxide, and efficient heat and water management [1,2].

Extensive research has been ongoing towards the development of polymer electrolyte membranes with strong proton conductivity under anhydrous conditions along with chemical, thermal, and electrochemical stability. Studies in this area include the incorporation of inorganic proton conductive materials to prepare composite membranes from the commonly used perfluorosulfonic acid membranes and polysulfone (PS), polybenzimidazole (PBI), poly(ether ether ketone) (PEEK), and their sulfonated counterparts [3,4,5,6]. Acid-doped basic polymeric membranes such as PBI doped with phosphoric acid have also been studied extensively for this purpose.

Ionic liquids (ILs), also known as ionic salts, have been extensively utilized for various applications such as in separation processes, polymerization, organic synthesis, and electrochemical reactions. ILs offer very attractive properties such as good ionic conductivity along with low vapor pressure and good electrochemical, chemical, and thermal stability. These properties make ILs a material of choice for utilizing them as dopants in the development of composite PEMs [7,8].

Methanesulfonic acid is a strong oxoacid (pKa −1.92) [9] and is a relatively stable, halogen-free, readily biodegradable compound with low toxicity, making it an environmentally benign choice for PIL synthesis for such applications [10]. Furthermore, lower overpotentials have been reported for hydrogen oxidation and oxygen reduction for oxoacid-based protic ionic liquids [11,12]. These potential advantages have prompted us to study methanesulfonic-acid-based, protic-ionic-liquid-based polymer composite membranes under anhydrous conditions as potential electrolytes for high-temperature PEMFCs. Kreuer et al. [13] showed that the addition of basic molecules such as imidazole to oxoacids leads to an improvement in the ionic conductivity of the system. We, therefore, also studied the effect of the addition of excess base in the composite PEMs.

## 2. Materials and Methods

### 2.1. Materials

Methanesulfonic acid (MSA) (≥99.0%), triethylamine (TEA) (≥99.5%), N-methylpyrrolidine [NMP] (99%), and Tetraethylenepentamine (TEPA) (technical grade) were procured from Sigma-Aldrich. Ethylamine (EA) (99%) and diethanolamine (DEA) (98% for synthesis) were procured from Panreac, AppliChem, GmbH, Germany. All chemicals were used as received without any further purification.

### 2.2. Methods

#### 2.2.1. Synthesis of Methanesulfonic-Acid-Based Ionic Liquids

**Scheme 1:**
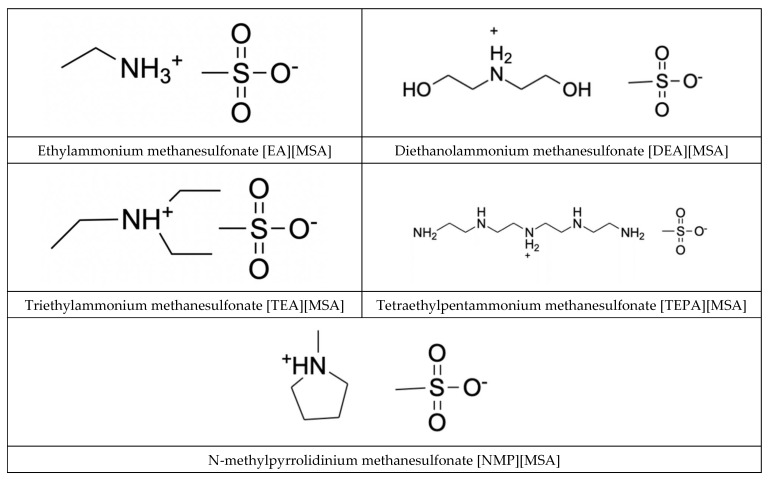
Structures of the methanesulfonic-acid-based protic ionic liquids synthesized in this work.

The synthesis was carried out in a two-necked, round-bottomed flask fitted with a thermometer and gas inlet for purging nitrogen gas. The reaction was carried out in an ice bath to manage the heat generated during the reaction. The reaction occurred under a nitrogen atmosphere and without any solvent; using a spatula, the acid MSA (0.2 mol, 19.23 g) was added gradually to 0.2 mol of the base with constant stirring (Equation (1)). The mixture was vacuum-dried at 90 °C for 8 h and stored for further analysis. The structural formula of the synthesized ionic liquids is shown in Scheme 1.

B + H-A → [H-B]^+^[A]^−^ (1)

[EA][MSA]: Methanesulfonic acid (0.2 mol, 19.3 g) and ethylamine (0.2 mol, 89.08 g) at ambient temperature (27 °C) obtained a product that was a brown-color liquid at room temperature (yield: 83.98%).

[DEA][MSA]: Methanesulfonic acid (0.2 mol, 19.23 g) and diethanolamine (0.2 mol, 21.02 g) at ambient temperature (27 °C) obtained a product that was a light-yellow liquid at room temperature (yield: 92.50%).

[TEA][MSA]: Methanesulfonic acid (0.2 mol, 19.3 g) and triethylamine (0.2 mol, 20.201 g) at ambient temperature (27 °C) obtained a product that was a wine-color liquid at room temperature (yield: 73.71%).

[NMP][MSA]: Methanesulfonic acid (0.2 mol, 19.3 g) and N-methylpyrrolidine (0.2 mol, 17.03 g) at ambient temperature (27 °C) obtained a product that was a light-yellow-color liquid at room temperature (yield: 98.54%).

[TEPA][MSA]: Methanesulfonic acid (0.2 mol, 179.3 g) and tetraethylenepentamine (0.2 mol 39.86 g) at ambient temperature (27 °C) obtained a product that was a yellowish semisolid at room temperature (yield: 75.51%).

#### 2.2.2. Polymer Electrolyte Membrane Fabrication

A homogeneous solution of DMAc containing 0.1 wt.% of LiCl as a stabilizer was prepared by dissolving 100 mg of LiCl in 100 mL of DMAc by stirring for 1 h at room temperature. A PBI solution of 10 wt.% was prepared by dissolving PBI powder in the DMAc solution by heating it overnight at 120 °C with stirring. The PBI membranes were prepared via the solution-casting method as this widely used technique ensures consistent and reproducible membrane fabrication. The PIL, diethanolammonium methanesulfonate [DEA][MSA], and different mole % of the excess base (diethanolamine) were added to the PBI solution and mixed by stirring and then cast onto a clean glass petri dish and dried at 80 °C for 48 h. The membranes were washed with distilled water and dried under vacuum at 80 °C in order to remove any residual solvent and the stabilizer. The thicknesses of the prepared membranes were between 240 and 280 μm.

#### 2.2.3. FTIR Spectroscopy

FTIR spectroscopy was used for the vibrational characterization of all the PILs on the basis of interactions between the methanesulfonic acid and the different nitrogen-containing bases. The full range of infrared, typically used to characterize the organic molecules, was covered during the analysis of the prepared samples. A Perkin Elmer FTIR spectrophotometer (Spectrum 100, Perkin Elmer Cetus Instrument, Norwalk, CT, USA) was used to obtain the FTIR spectra of the above samples on a KBr cell at room temperature in the 4000–400 cm^−1^ frequency region. The FTIR spectra of the neat PBI and the composite membrane samples were obtained in ATR mode at room temperature using a Nicolet iN10 FTIR (Thermo Scientific, Winsford, UK) with a germanium microtip accessory in the 4000–650 cm^−1^ frequency region.

#### 2.2.4. ^1^H and 13C NMR Analysis

Nuclear magnetic resonance (NMR) ^1^H NMR and ^13^C-NMR spectra were obtained for the synthesized PILs using an NMR spectrometer (JEOL DPX400MHz, Tokyo, Japan). Deuterated chloroform (CDCl_3_) was used as a solvent for the preparation of samples and tetramethylsilane (TMS) as the internal standard.

#### 2.2.5. Differential Scanning Calorimetry (DSC)

A differential scanning calorimeter (DSC-60A, Shimadzu, Tokyo, Japan) was used to analyze the melting and crystallization behaviors of the protic ionic liquids and the composite PEMs. To obtain the exothermic/endothermic crystallization/melting curves, the PIL samples were heated from room temperature to 150 °C at a heating rate of 10 °C per minute, then held at 150 °C for 10 min to stabilize the system and avoid drifting due to the addition of liquid nitrogen to the refrigerant reservoir; it was then cooled to −50 °C and again allowed to stabilize for 10 min before the recording of the second heating and cooling cycle. The composite PEMs were also subjected to DSC analysis in a similar fashion from 25 to 500 °C. All the DSC measurements were made in a nitrogen environment.

#### 2.2.6. Thermogravimetric Analysis (TGA)

Utilizing a thermogravimetric analyzer (Mettler Toledo AG, Analytical CH-8603, Schwerzenbach, Switzerland), we studied the thermal stability of the ionic liquids and the composite PEMs in an argon atmosphere (at a flow rate of 50 mL/min). The samples (8–10 mg) were heated in an alumina pan at a heating rate of 10 °C/min from room temperature to 650 °C (held at 110 °C for a period of 30 min. to quantify the amount of water present in the samples).

#### 2.2.7. Scanning Electron Microscopy (SEM)

The cross-section morphology of the cryo-fractured membranes was studied using scanning electron microscopy. The samples were coated with gold using a Quorum Q150R S sputter coater (Quorum Technologies Ltd., Laughton, UK) at 20 mA for 90s. The samples were then visualized using a ZEISS EVO LS 10 scanning electron microscope (Carl Zeiss Microscopy GmbH, Jena, Germany). Images were captured using the ZEISS SmartSEM version 5.05 software (Roduit, N., Geneva, Switzerland).

#### 2.2.8. Electrochemical Impedance Spectroscopy (EIS)

**Figure 1:**
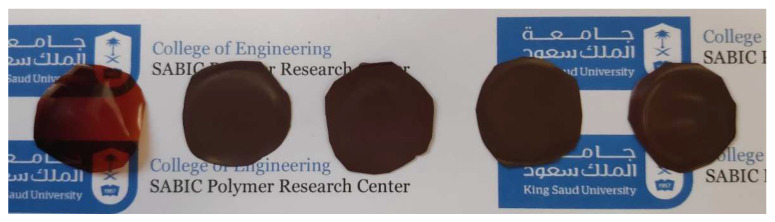
Photographs of PIL-based composite PEMs (from left to right) Neat PBI, PBI-50PIL-0DEA, PBI-50PIL-25DEA, PBI-50PIL-50DEA, and PBI-50PIL-100DEA.

EIS was used to measure the ionic conductivity of the PILs in the temperature range of 120 °C to 65 °C. Prior to the ionic conductivity measurements, the PIL was dried overnight by heating it at 125 °C. A Teflon spacer was used to create space between two platinum plates (the blocking electrode) to accommodate the ionic liquid sample for the measurements [14]. The membrane samples (Figure 1) were sandwiched between stainless steel blocking electrodes for the EIS measurements. The samples were tested using a Palmsens4 Impedance Analyzer (PalmSens BV, Houten, The Netherlands) to measure the real and imaginary impedances from 100 kHz to 10 Hz, with an alternating current of 20 mV amplitude. The bulk resistance (R_b_) was estimated from the high-frequency intercept of the Nyquist plots. We used the formula = σ = l/(A R_b_), where l is the membrane thickness and A is the area of the membrane sample.

## 3. Results and Discussion

### 3.1. FTIR Spectroscopy

**Figure 2:**
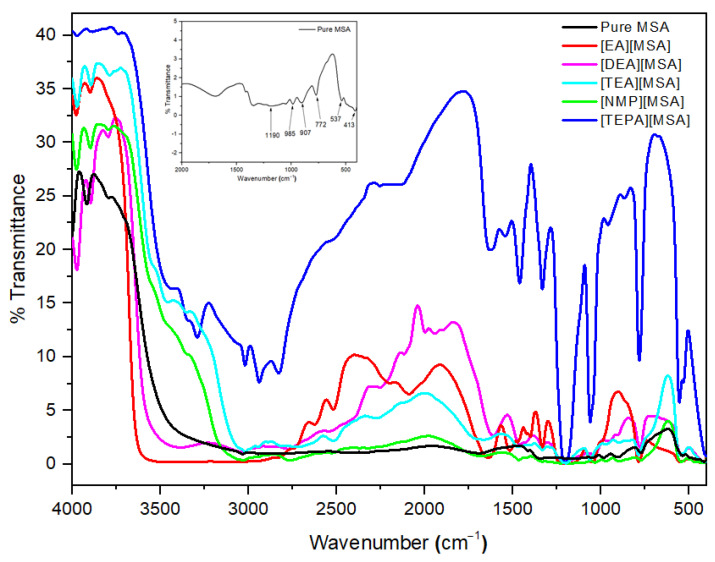
FTIR spectra of MSA and the synthesized protic ionic liquids.

**Figure 3:**
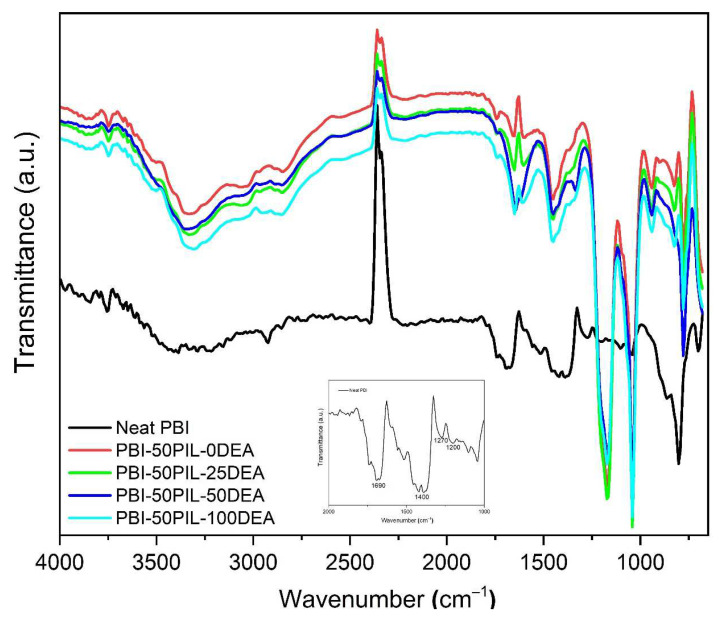
FTIR spectra of neat PBI and the synthesized composites.

The FTIR spectrum (Figure 2) shows the presence of the specific peaks of methanesulfonic acid, which can be assigned to its different active components; the peaks at 1190 cm^−1^ and 985 cm^−1^ can be assigned to the asymmetric and symmetric stretching of SO_3_. The S-OH [15] and the C-S stretching peaks were obtained at 907 cm^−1^ and 772 cm^−1^, respectively [16,17]. The peaks for SO_3_ bending and rocking were obtained at 537 cm^−1^ and 413 cm^−1^, respectively [16]. The spectra of various PILs prepared in this study are shown in Figure 3. When the peaks of methanesulfonic acid were comparatively studied in the prepared PILs, it was observed that they showed significant shifts in the assigned values, which indicates the formation of the salts due to the combination of methanesulfonic acid and the different bases used. As most of the peaks shifted and had a slightly broadened nature (2200 cm^−1^–3200 cm^−1^), it may be suggested that these also developed hydrogen bonds among them. It may also be observed that a broadened peak around 1340 cm^−1^ is present in the salt samples which can be attributed to the interactions of the sulfonate ions [18]. It may also be suggested from the spectra that the samples were moisture-free as there is no significant peak for OH in the frequency range 3400 cm^−1^–3500 cm^−1^. Furthermore, from the above discussion, it is safe to conclude the successful formation of the salts of methanesulfonic acid with the different bases.

The important peaks of imidazole for isolated N-H stretching were reported around 3400–3500 cm^−1^, while the peaks for the self-associated N-H bonds were found in the range of 2500–3400 cm^−1^. These bands may be broad if there is the presence of detectable moisture. This may be seen in Figure 3, where the above specified peaks are present; however, their broad shape suggests the presence of a detectable level of moisture in the sample. It can also be suggested that the humidity was higher than the required limit during the analysis. The peak of the N^+^-H vibrations (protonated imine in the doped samples) was also reported to be present within the range of the above broad peak region. The peaks for the C=N stretching vibration are present in the 1690 cm^−1^ region [19,20]. The band around 1400 cm^−1^ can be attributed to the in-plane deformation of the benzimidazole rings. The in-plane C-H deformations appear around 1200 cm^−1^. The peak at 1270 cm^−1^ can be assigned to the breathing mode of the imidazole ring in PBI [20,21].

Upon the addition of DEA in the PBI film, it may be observed in Figure 3 that most of the above-discussed peaks in PBI are present; however, the intensity either decreased or the peaks broadened due their interaction with DEA. It may also be seen that the specified peaks of DEA in the composites slightly shifted from their original positions, indicating their interactions with PBI.

### 3.2. ^1^H and ^13^C NMR Chemical Shifts

**Figure 4:**
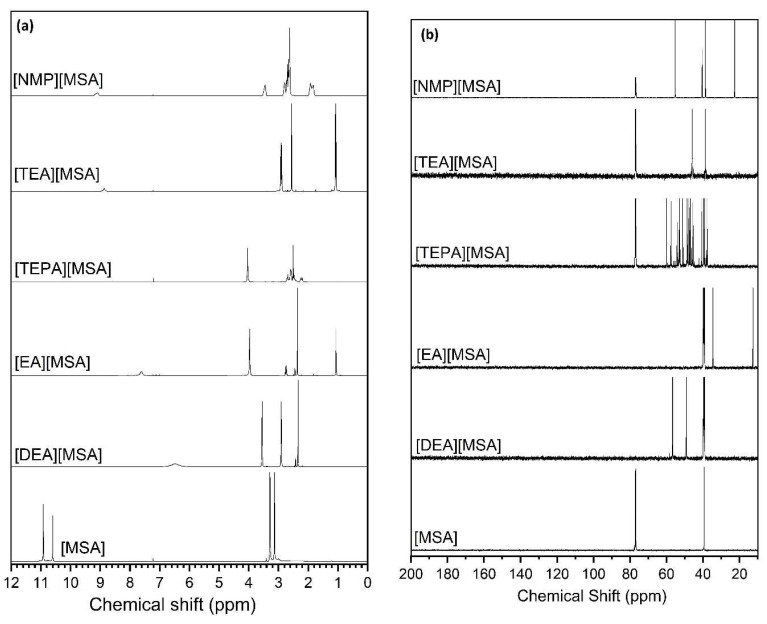
(**a**). ^1^H spectra (**b**) ^13^C NMR of the PILs.

Figure 4 a and b show the ^1^H and ^13^C NMR spectra of the PILs, respectively. The PILs are formed by the substitution of the proton of the respective bases with different cations; the ^1^H spectra of the PILs exhibit a chemical shift relative to the pure MSA, as shown in Figure 4a. Figure 4b displays the ^13^C NMR results, which also show a consistent shift in the spectra of the PILs in comparison to that seen in the pure MSA.

### 3.3. Thermal Behavior (DSC)

Ionic liquids (ILs) are known to show peculiar thermal behavior due to the presence of a complex packing of cations and anions [22]. Some ILs exhibit the formation of polymorphs, glasses, and liquid crystals [23,24]. The crystallization and glass transition phenomena are kinetically driven and depend on the cooling/heating rate and thermal history of the sample. Therefore, a large variation in the reported T_c_ and T_g_ values of the ILs can be found in the literature [25,26]. However, the melting point (T_m_) values can be obtained rather accurately. Small variations in T_m_ values can be ascribed to the presence of water and/or other impurities.

**Figure 5:**
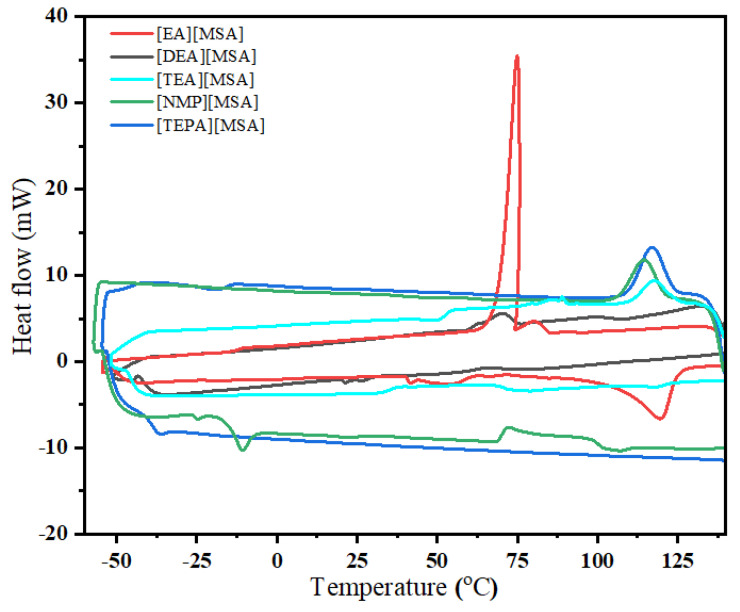
DSC thermogram of the methanesulfonate-anion-based protic ionic liquids.

The DSC thermograms and related data of the PILs are presented in Figure 5 and Table 1, respectively. The thermal data for only two of the PILs, i.e., [EA][MSA] and [TEA][MSA] are available in the literature. [EA][MSA] had the lowest amount of water present among the PILs. It did not show a glass transition; rather, it showed a melting point around 115 °C. This value is in good agreement with the one reported earlier by Beliers and Angell [26], where a similar amount of water was present in the sample. 

The case of [TEA][MSA] is rather complex. There are discrepancies in the reported thermal behavior of this PIL. Earlier reports show that [TEA][MSA] is a glass former [11,27,28,29] and shows a T_g_ in the range of −62 °C to −97 °C. Also, the melting point for this PIL was reported in the range of 17 °C to 33 °C. The variation in these values could be due to the presence of different amounts of water (for some samples, the water content was not reported). Recent reports reveal that this IL is not a glass former; rather, it just shows a melting phenomenon in the range of 25 °C to 33 °C [29,30,31]. In this work, we also observed the absence of a glass transition and a melting temperature of around 31 °C, which is in good agreement with the values reported in the literature [29,30,31].

The PIL [TEPA][MSA] did not show any melting; rather, it just exhibited a glass transition temperature around −35 °C. This result is not surprising as this IL is highly viscous. The other two PILs, [DEA][MSA] and [NMP][MSA], showed melting peaks around 22 °C and −10 °C. The methanesulfonate salts were reported to exhibit broad melting peaks [33] with classical melting behavior, as observed in this work.

**Figure 6:**
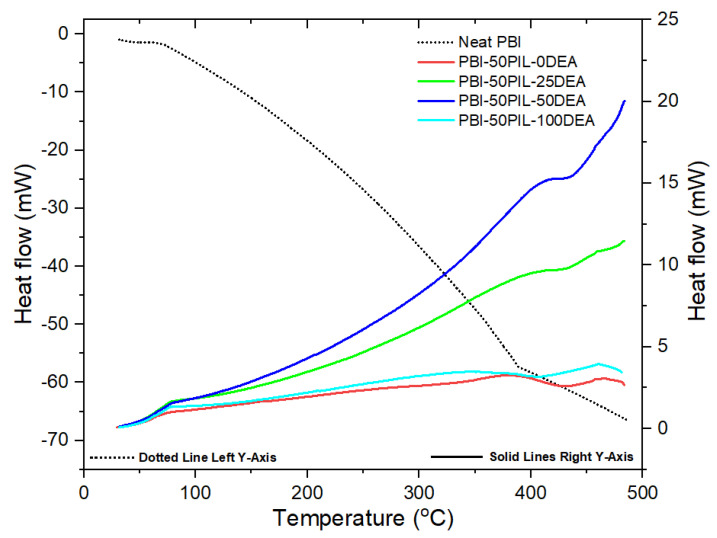
DSC thermogram of the PIL-based PBI composite PEMs.

The prepared PBI membranes with varying amounts of PILs ([DEA][MSA]) were also subjected to thermal analysis (Figure 6). One can clearly observe that the neat PBI showed a glass transition temperature of around 400 °C, which showed a downward shift with the incorporation of the PILs. The incorporation of the PIL and the excess base had a plasticizing effect on the PBI polymer matrix. Similar behavior was also reported earlier for PBI membranes with other PILs [34].

### 3.4. Thermogravimetric Analysis

**Figure 7:**
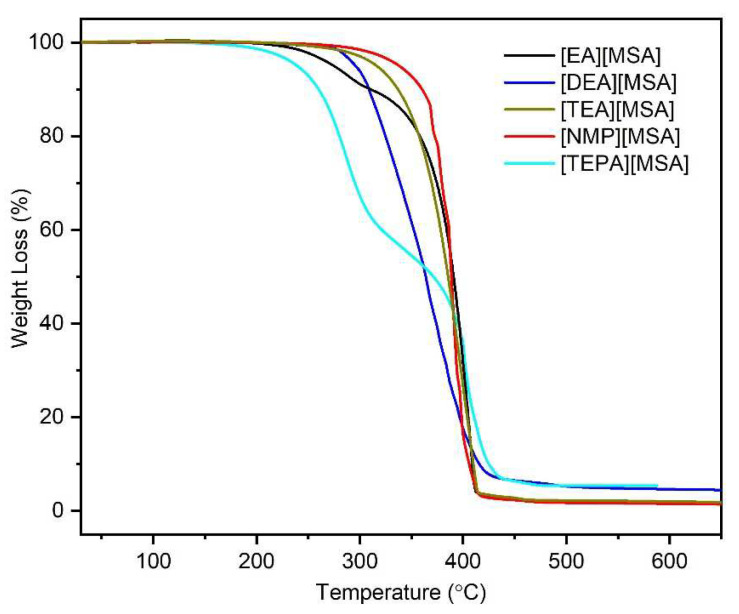
TGA thermogram of the methanesulfonate-anion-based protic ionic liquids.

Moreover, the thermal stability of the PILs was measured using thermogravimetric analysis (Figure 7). Some of the PILs were hygroscopic; therefore, the PILs were held at 110 °C for a period of 30 min. The quantification of the water present in the samples can be performed using the weight loss while holding the sample at 110 °C. A negligible amount of water was observed in [EA][MSA], whereas [DEA][MSA], [TEA][MSA], [TEPA][MSA], and [NMP][MSA] contained 0.5 wt.%, 1.5 wt.%, 1.5 wt.%, and 0.75 wt.% water, respectively. The onset of decomposition temperatures (T_onset_) of the PILs, where the first sign of weight loss was observed, was in the range of 213 °C to 315 °C. All the ILs showed complete dissociation in a single step, except for [TEPA][MS], which exhibited a two-step dissociation curve over the studied temperature range. The highest decomposition temperature was observed for [DEA][MSA] followed by [NMP][MSA], [EA][MSA], [TEA][MSA], and [TEPA][MSA], respectively. The decomposition temperature of [EA][MSA] and [TEA][MSA] agreed well with the one reported in the literature within the experimental uncertainties. 

**Figure 8:**
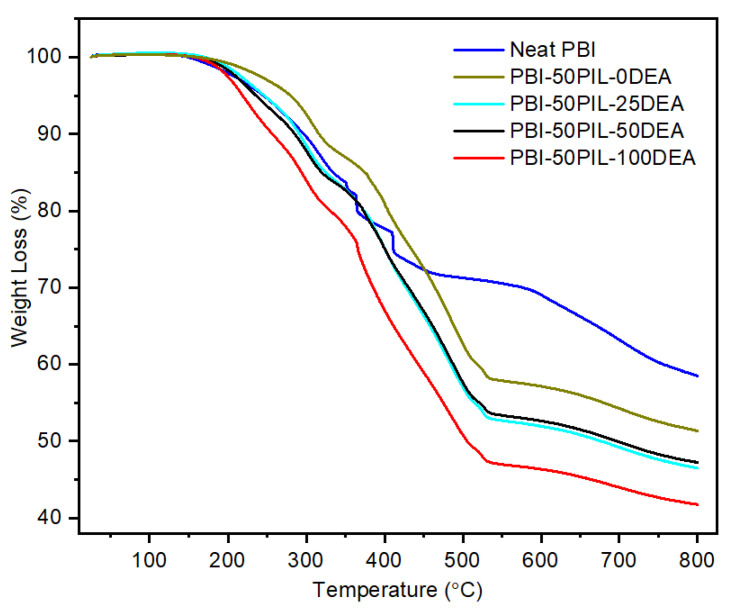
TGA thermogram of the PIL-based PBI composite PEMs.

The thermal stabilities of the composite PBI membranes with varying amounts of excess amine were also studied in order to understand the optimum blending concentration (Figure 8). In comparison to the neat PBI, PBI-50PIL-0DEA showed a relatively higher onset of decomposition temperature, whereas the further increase in DEA slightly brought down the onset of decomposition temperatures. The degradation curves followed a two-step decomposition pattern, as observed elsewhere [34,35]. The first degradation started around 250 °C, which was mainly due to the onset of the degradation of the PILs, as observed in the thermal stability results for the PILS. The second degradation was observed around 400 °C, which was due to the degradation of PBI polymer backbone chains. The thermal stability of the membrane decreased slightly, but still, it is good enough for the membranes to be used for high-temperature applications. 

### 3.5. Scanning Electron Microscopy

**Figure 9:**
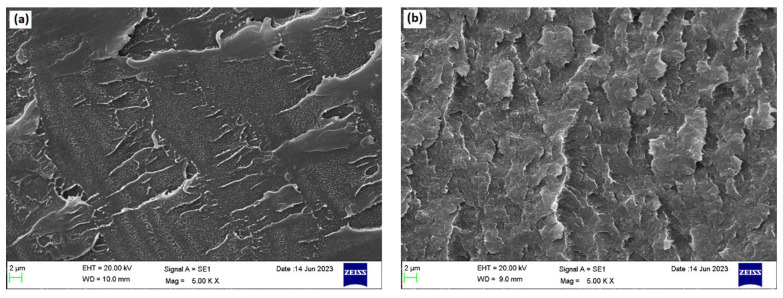

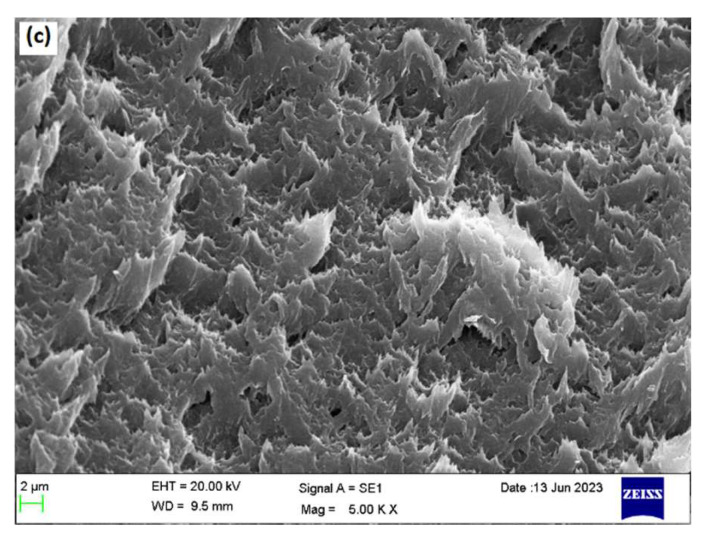
SEM images of (**a**) neat PBI membrane, (**b**) PBI-50PIL-0DEA membrane, and (**c**) PBI-50PIL-50DEA membrane.

The morphologies of the cryo-fractured cross-section of the membranes were studied using SEM. The SEM images of these cryo-fractured cross-sections of the neat PBI and the composite membranes are shown in Figure 9. The cross-section morphology of the neat PBI membranes shows a clean cryofracture with a dense structure free of porosity. The addition of the PIL to the PBI polymer matrix led to the formation of a porous structure due to the formation of channels, owing to the presence of the PILs in the polymer network. As evident from the SEM images, the addition of excess DEA led to a further increase in the porosity of the membranes. These observations are consistent with results reported earlier for similar systems [36].

### 3.6. Electrical Transport Properties

**Figure 10:**
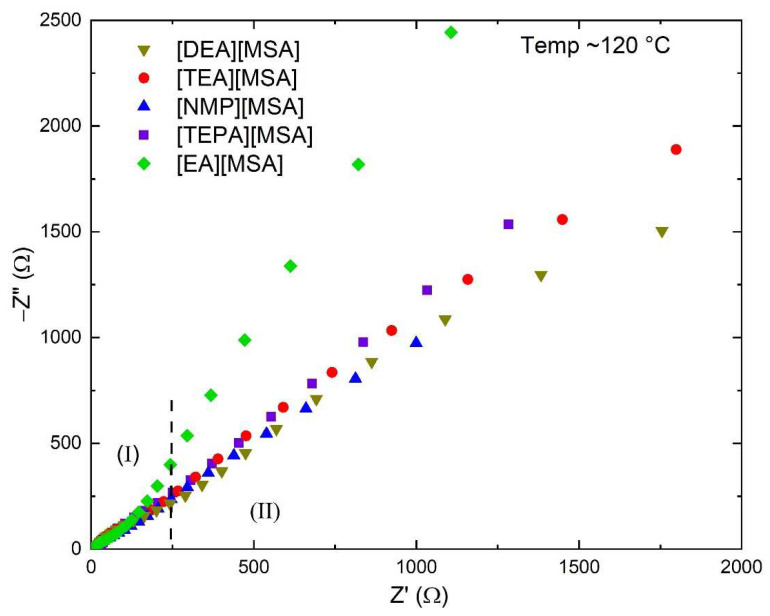
Nyquist plots for [MSA]-based PILs with different cations.

As mentioned earlier, ILs are known to have ions as charge carriers. Electrochemical impedance spectroscopy is widely used to determine the transport properties of ionic conductors. Figure 10 shows the Nyquist plots for the [MSA]-based PILs with different cations at ~120 °C. Depicted is an undeveloped semicircle in Region—I (high-frequency domain) and a straight line in Region—II (low-frequency domain) [37]. The former corresponds to the ionic diffusion phenomenon and the latter is due to the blocking-electrode effect. The curve was fitted using the Debye circuit model with a constant-phase element. The fitting yielded the bulk resistance (*R*_b_), and thereby the electrical conductivity (σ) of the PIL. The PILs exhibited similar values of σ_120°C_: 1.5 × 10^−3^ S/cm for [TEPA][MSA]; 1.6 × 10^−3^ S/cm for [DEA][MSA]; and 1.7 × 10^−3^ S/cm for [TEA][MSA], while they exhibited a relatively higher value of σ_120°C_: 3.5 × 10^−3^ S/cm for [EA][MSA] and 6.9 × 10^−3^ S/cm for [NMP][MSA]. As revealed by the DSC study, the higher values of σ_120°C_ for PILs with EA and NMP were due to the liquid state of the PILs, offering easy ion transport.

**Figure 11:**
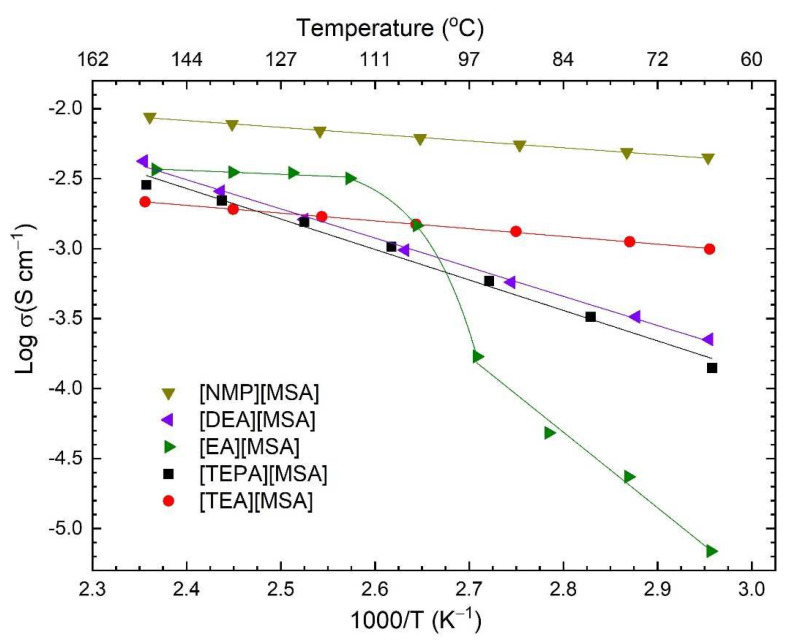
Log σ - T^–1^ curve for [MSA]-based PILs with different cations.

Temperature-dependent study of electrical conductivity is required to evaluate the activation energy (E_a_) of the PILs for knowing the easiness of ion transport. Figure 11 shows the temperature dependence of electrical conductivity for the methanesulfonate-based PILs with different cations. The PILs exhibited a linear log σ - T^−1^ curve, revealing Arrhenius-type behavior with an equation, σ = σ_o_ exp [−E_a_/k_B_T], where the notations have their usual meanings [14]. The PIL [EA][MSA] had linear curves before and after the melting temperature. We calculated the value of E_a_ from the slope of the log σ - T^−1^ plot, which is as follows: 0.11 eV for [TEA][MSA], 0.10 eV for [NMP][MSA], 0.41 eV for [DEA][MSA], 0.43 eV for [TEPA][MSA], and 1.07 eV and 0.05 eV before and after the melting temperature of [EA][MSA], respectively. For a device application, an E_a_ value lower than 0.3 eV is required [38].

**Figure 12:**
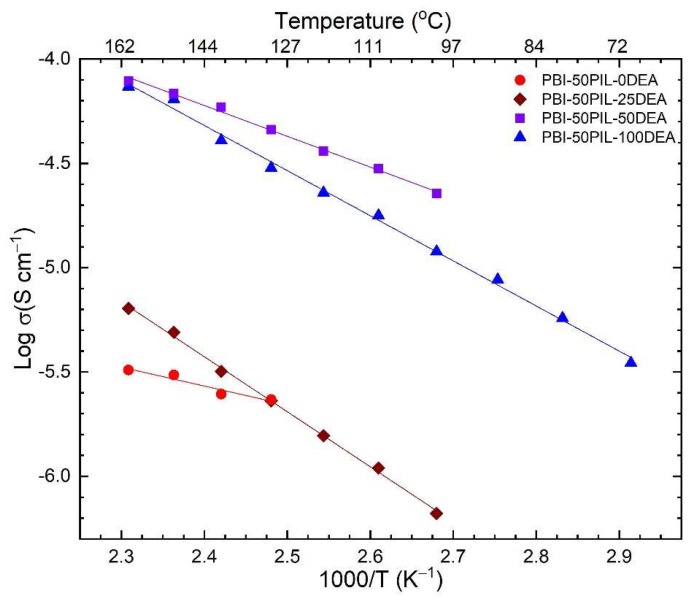
Log σ - T^–1^ curve for the composite PEMs.

The PEMs were prepared by incorporating a [DEA][MSA] PIL 50% by weight in the PBI polymer matrix via the solution-casting method. The PILs [TEA][MSA], [NMP][MSA], [TEPA][MSA], and [EA][MSA] were incompatible with the PBI matrix. These PILs either precipitated in the polymeric solution or phase-separated during the drying process. Only the [DEA][MSA] showed good compatibility with the PBI, and a relatively better electrical conductivity was achieved for 50 wt. % content of the PIL. We also incorporated a different mole percent of the corresponding amine [DEA] in the composite electrolyte to analyze if the presence of excess amines helped in increasing the proton-conducting pathways in the membranes. The composite PEMs with 50 wt. % PIL content and 0, 25, 50, and 100 mol % excess amine were denoted as PBI-50PIL-0DEA, PBI-50PIL-25DEA, PBI-50PIL-50DEA, and PBI-50PIL-100DEA, respectively. The incorporation of excess acid (MSA) in the composite PEMs was not feasible since any amount of excess (MSA) was precipitated the PBI polymer solution. We observed σ_130°C_ values of 2.47 × 10^−6^, 2.18 × 10^−6^, 4.3 × 10^−5^, and 2.8 × 10^−5^ S/cm for the PBI-50PIL-0DEA, PBI-50PIL-25DEA, PBI-50PIL-50DEA, and PBI-50PIL-100DEA PEMs, respectively. The temperature dependence of the proton conductivity for these membranes is shown in Figure 12. The membranes showed a linear log σ - T^−1^ curve for all the PEMs, revealing the Arrhenius nature of the electrolytes, with E_a_ values of 0.17 eV, 0.52 eV, 0.29 eV, and 0.42 eV for the PEMs with 0, 25, 50, and 100 mol % excess amine, respectively. The membrane with 50 mol. % excess amine (PBI-50PIL-50DEA) is hereafter referred to as the optimum polymer-conducting electrolyte membrane due to its high electrical conductivity along with the low activation energy, and can be utilized for high-temperature fuel cell applications. One can also note that the membrane without any excess amine (PBI-50PIL-0DEA) showed electrical conductivity only at higher temperatures (after 125 °C); however, the incorporation of the excess amine granted ionic conductivity to the PEMs at lower temperatures as well.

## 4. Conclusions

Methanesulfonate-oxoacid-based PILs were successfully synthesized and characterized to evaluate their thermal and physical properties for their potential application in electrochemical devices. FTIR and NMR studies confirmed the formation of the PILs. The thermal properties of the PILs were investigated through DSC and TGA studies. The change from a dense to a porous morphology for the composite membranes was observed from SEM images. The proton conductivity of the PILs was evaluated using EIS and was found to be in the order of 10^−3^ S/cm for all the PILs. The PILs exhibited a linear log σ - T^−1^ curve, revealing the Arrhenius type of temperature-dependent behavior. The diethanolammonium methanesulfonate PIL was incorporated in a PBI polymer matrix along with different mole percents of excess amine to successfully fabricate a composite PEM for high-temperature application. The incorporation of the excess amine augmented the proton conductivity of the composite PEMs and showed Arrhenius-type temperature-dependent behavior. The composite PEM with 50 mol % excess incorporated base showed the optimal properties for prospective device applications.

## Figures and Tables

**Table 1:** Thermal properties of the methanesulfonate-anion-based protic ionic liquids (PILs).

Ionic Liquid	T_m_ (°C)	T_g_ (°C)	T_dec_	H_2_O Content
Ethylammonium methanesulfonate [EA][MSA]	114.85 112.5 [27]	na na	264.0 288.6 [27]	~0 wt% <1 ppm [27]
Diethanolammonium methanesulfonate [DEA][MSA]	21.79	na	291.8	0.5 wt%
Triethylammonium methanesulfonate [TEA][MSA]	31.1 21.6 [12] 33.1 [13] 33.0 [14] 25.0 [15] 24.3 [16] 33.0 [17] 17.4 [19]	na −96.5 [27] na [28] na [29] na [30] −78.9 [31] −75.0 [32] −62.1 [11]	247.5 269.7 [27] 203.0 [28] 305.0 [29] 218.0 [30] 290.0 [32] 225.0 [11]	1.50 wt% <1 ppm [27] 0.81 wt% [28] 50 ppm [29] 2 mol% [30] 100 ppm [31]
Tetraethylpentammonium methanesulfonate [TEPA][MSA]	na	−35.32	163.5	1.50 wt%
N-methylpyrrolidinium methanesulfonate [NMP][MSA]	−10.15	na	285.0	0.75 wt%

## Data Availability

Data are available within the article.
